# Exploring the role of carers in psychiatric home treatment: qualitative multi-method study

**DOI:** 10.1186/s12888-026-08018-9

**Published:** 2026-04-01

**Authors:** Emma Kula, Marie Salzmann, Tamara Waldmann, Konstantinos Nikolaidis, Gerhard Längle, Peter Brieger, Jürgen Timm, Lasse Fischer, Svenja Raschmann, Martin Holzke, Sandeep Rout, Constance Hirschmeier, Johannes Hamann, Uwe Herwig, Johanna Baumgardt, Reinhold Kilian, Stefan Weinmann, Andreas Bechdolf, Sebastian von Peter, Julian Schwarz

**Affiliations:** 1 Faculty of Health Sciences Brandenburg, Brandenburg Medical University Theodor Fontane, Neuruppin, Germany; 2 Department of Psychiatry and Psychotherapy, Center for Mental Health, Immanuel Hospital Rüdersdorf, Brandenburg Medical School Theodor Fontane, Seebad 82/83, 15562 Rüdersdorf, Germany; 3 Section of Health Economics and Health Services Research, Department of Psychiatry and Psychotherapy II, Ulm University at Bezirkskrankenhaus Günzburg, Günzburg, Germany; 4 Department of Psychiatry, Psychotherapy, and Psychosomatics incorporating FRITZ am Urban and soulspace, Vivantes Hospital am Urban and Vivantes Hospital im Friedrichshain, Berlin, Germany; 5 Department of Psychiatry and Psychotherapy, Campus Charité Mitte, Charité - Universitätsmedizin Berlin, corporate member of Freie Universität Berlin and Humboldt-Universität zu Berlin, Berlin, Germany; 6 Department of Psychiatry and Psychotherapy Zwiefalten, Center for Psychiatry South Württemberg, Zwiefalten, Germany; 7 Clinic for Psychiatry and Psychosomatics of Reutlingen (PP.rt), Academic Teaching Hospital of the University of Tübingen, Reutlingen, Germany; 8 kbo-Isar-Amper Hospital Munich Region, Academic Teaching Hospital of Ludwig-Maximilians-University Munich, Haar near Munich, Germany; 9 Competence Center for Clinical Studies Bremen, Biometrics Department, University of Bremen, Bremen, Germany; 10 Center for Psychiatry South Württemberg, Department of Psychiatry and Psychotherapy I, University of Ulm, Weissenau, Germany; 11 Department of Psychiatry, Psychotherapy, and Psychosomatics, Vivantes Neukölln Hospital, Berlin, Germany; 12 Mainkofen District Hospital, Deggendorf, Germany; 13 Reichenau Center for Psychiatry, Academic Teaching Hospital University of Konstanz, Reichenau, Germany; 14 Psychiatric University Hospital Zurich, Zurich, Switzerland; 15 Scientific Institute of the AOK (WIdO), Berlin, Germany; 16 University Psychiatric Clinics (UPK), University of Basel, Basel, CH Switzerland; 17 Center of Integrativ Psychiatry, Department of Psychiatry, Psychotherapie, and Psychosomatics, University of Luebeck, Luebeck, DE Germany

**Keywords:** Family carers, Relatives, Home treatment, Qualitative research, Caregiver burden, Mental health services

## Abstract

**Background:**

This qualitative study explored the roles, experiences, and needs of informal carers such as family and close friends in acute psychiatric home treatment in Germany.

**Methods:**

Data was collected through individual interviews, focus groups, and participant diaries with 27 carers and analyzed using grounded theory methodology.

**Results:**

The findings highlight the central yet multifaceted role carers play in facilitating care - initiating treatment, providing emotional containment, supporting daily functioning, and acting as intermediaries between service users and professionals. However, these roles were often poorly defined, leading to uncertainty, role conflicts, and occasional exclusion from the treatment process. Limited or unclear communication about expectations, responsibilities, and boundaries exacerbated this ambiguity. Carers also reported significant emotional and practical burdens, sometimes reaching a level of strain that jeopardized the continuation of home treatment. Recognition of their contributions, proactive engagement by the treatment team, and tailored support strategies were identified as key facilitators of positive involvement.

**Conclusions:**

The results underscore the need to systematically embed role clarification, structured collaboration, and carer support into the design and delivery of home treatment services. Future research should further explore how explicit clearly defined role negotiation and early joint planning meetings can enhance carer involvement while mitigating strain.

**Trial registration:**

German Clinical Trials Register (DRKS), DRKS00022476. Registered December 3^rd^ 2020, https://drks.de/search/de/trial/DRKS00022476; ClinicalTrials.gov, Identifier: NCT0474550. Registered February 9th 2021.

**Supplementary Information:**

The online version contains supplementary material available at 10.1186/s12888-026-08018-9.

## Introduction

Internationally, home treatment (HT) is increasingly being implemented as an alternative to inpatient care for people experiencing psychiatric crises [1]. Key characteristics of HT include mobility, the provision of care in the service user’s own home, and the systematic involvement of carers and the broader social network [2]. By working in the familiar home environment, outreach-based psychiatric treatment models can identify and address both resources and barriers to recovery at an early stage [3]. Such approaches have been shown to reduce hospital stays while maintaining treatment effectiveness, allowing service users to remain connected to their daily routines and social contexts [4].

Unlike in inpatient settings, where informal carers are often involved only sporadically through occasional family meetings, HT creates opportunities for ongoing, everyday-oriented participation of close contacts [5]. There is extensive evidence that structured involvement of carers, e.g. through psychoeducation, shared decision-making, or supportive interventions, can both reduce their own distress and improve clinical outcomes for service users [6–9]. This proximity can strengthen collaboration, improve information flow, and support recovery. However, it can also blur the boundaries between professional and informal care, potentially shifting greater responsibility to carers.

Regardless of the treatment setting, carers of people with mental illness provide substantial emotional, practical, and sometimes physical support [10]. Mental illness can place a significant burden not only on the affected person but also on their social environment. Carers are at increased risk of developing mental health problems themselves, particularly depression and anxiety disorders, and often have their own unmet support needs [11, 12]. There is even data suggesting that caregivers are at high-risk for suicide [13]. At the same time, their contributions substantially relieve formal healthcare systems with an estimated economic value of approximately €35,000 to €125,000 per person annually [14].

There is conflicting evidence regarding the reduction of caregiver burden in HT. This may result from wide international variation in the implementation of HT. International findings suggest that intensive outreach models such as Assertive Community Treatment (ACT) can foster openness and cooperation with carers, leading to greater acknowledgment, information sharing, and involvement in decision-making, thereby improving their experiences compared with previous services [15]. However, there is also a risk of overburdening carers – especially when their roles are undefined or when adequate support structures are lacking [5, 16]. For some, the demands of HT may be as high as, or even higher than, during inpatient care, particularly in severe crises. A study on Crisis Resolution Home Treatment in Spain, while reporting a significant increase in service user satisfaction, did not show a reduction in caregiver burden [17]. In contrast, a qualitative study on community-based integrated community mental health care in Germany found that caregivers experienced the service as a significant relief, with a reduced sense of caregiving burden, as they did not feel left alone in facing challenges [18]. Likewise, a systematic review reported a significant reduction in caregiver burden six months after community crisis intervention [19]. In Norway, a majority of caregivers experience an open, cooperative approach from ACT teams, which they consider essential and an improvement compared with services in other settings [15].

Studies report higher levels of caregiver dissatisfaction with inpatient treatment, with one study indicating a 10% increase in caregiver burden compared with HT [20]. A perceived lack of quality of care for their loved ones as well as insensitivity and a lack of acknowledgment and information for caregivers may contribute to this, leading to a general sense of exclusion and alienation [21]. Nevertheless, in some cases, caregivers prefer hospitalization over HT. This may be related to a lack of support, as a preference for hospitalization has been associated with a greater perceived burden related to the service user’s condition and less confidence in one’s own ability to cope [16].

Despite the growing implementation of HT in many countries, the role of carers in this setting remains insufficiently analyzed [22]. While there is a robust body of literature on caregiver burden, support needs, and the benefits of involvement in other psychiatric care contexts [23], corresponding studies in the context of intensive home-based psychiatric care are scarce. Initial qualitative findings suggest that caregivers in HT may perceive themselves as bearing primary responsibility for care, often with limited support, particularly in acute situations [16, 24].

Against this background, the present study explores the contributions, competencies, roles, and potential conflicts experienced by carers in the context of HT. In doing so, it aims to provide a more systematic understanding of carers’ perspectives, inform service development, and support the development of models that address the needs of both service users and their informal carers.

## Methods

### Setting

Currently, around 75 hospitals in Germany have implemented a specific form of intensive home treatment evaluated in this study, referred to as inpatient equivalent home treatment (IEHT) [41]. Compared to other HT models, IEHT ensures a particularly high level of treatment intensity by providing at least one daily face-to-face contact, mostly at the patient’s home, with a multiprofessional, team-based approach. Inclusion criteria for IEHT primarily comprise an indication for acute, otherwise inpatient psychiatric treatment and an adequate home that allows for privacy, evaluated by a psychiatrist, as well as the consent of all members of the household who are of legal age. Child safety and welfare also need to be considered [42]. Generally, all psychiatric conditions can be treated. Of the ten clinics across Germany that participated in this study with their IEHT teams, only one formally excludes patients who primarily suffer from addiction or could endanger themselves or others [25, 26, 43]. The study centers represented urban (*n* = 5), rural (*n* = 1), and mixed urban–rural (*n* = 4) catchment areas. Participating hospitals were located in Berlin (Vivantes Klinikum Am Urban, Vivantes Klinikum Neukölln, Charité – Universitätsmedizin Berlin), Brandenburg (Department of Psychiatry and Psychotherapy, Immanuel Klinik Rüdersdorf, affiliated with Brandenburg Medical School), Baden-Württemberg (Zentrum für Psychiatrie Südwürttemberg, including the Clinics for Psychiatry and Psychosomatics in Zwiefalten, Weissenau-Ravensburg, and Reutlingen, as well as the University Hospital of Psychiatry and Psychotherapy Tübingen, and the Zentrum für Psychiatrie Reichenau), and Bavaria (Kbo-Isar-Amper-Klinikum, Munich region).

### Participants

Informed consent was first obtained from service users participating in the overarching study to allow researchers to contact their carers for possible inclusion in this study. Eligibility criteria for carers included: (1) a trusting relationship, close kinship, or personal closeness to the service user; (2) explicit consent from the service user; and (3) sufficient proficiency in German or English. Carers were recruited across all ten study sites. While interviews and focus groups could be conducted after completion of HT, carers willing to keep a participant diary had to be identified at the beginning of the treatment period to ensure timely documentation. Given the well-documented challenges of recruiting carers in health research [44], no strict sampling criteria were applied. Instead, all eligible and available carers were invited until theoretical saturation was reached during data analysis [45]. For the stakeholder consultation, participants were approached via the national umbrella organization for carers of people with mental health conditions (BapK; https://www.bapk.de/*).* All participants provided written informed consent. They received financial compensation of 30€ for interviews or focus groups, 70€ for keeping a diary, and 100€ for stakeholder consultation. No prior relationship existed between researchers and participants. Only members of the research team were present during interviews or focus groups.

### Data collection

A literature search was conducted to explore the role of carers in mental health home treatment. Based on the findings, a semi-structured interview guide was developed (see Supplementary File 2) [46]. Due to the COVID-19 pandemic, all interviews (*n* = 13) were conducted by phone or video call, mainly by two researchers between March and August 2021. Interviews lasted one to two hours; one pre-test interview was included in the final sample. Interviewers documented observations in field notes, which were incorporated into the analysis. Sociodemographic data were also collected. All interviews were digitally recorded, transcribed, and anonymized.

A written guide for participant diaries was created (see Supplementary File 2), based on the central research question and gaps identified in the interview analysis. Participants documented their HT experiences over 28 days, either digitally or handwritten, between June and December 2022. They were encouraged to write daily and in close temporal proximity to treatment encounters. Weekly phone check-ins by researchers served as reminders and to clarify questions. Of eleven diaries distributed, eight were fully completed. All diaries were digitized, transcribed, and anonymized.

To address remaining knowledge gaps and validate preliminary findings, two focus groups were conducted between January and February 2023 using a shortened version of the interview guide. All carers invited to the overarching study were eligible. Eight participated, including two who had also taken part in earlier phases, allowing for further deepening of their perspectives. One focus group took place online and one at the Immanuel Hospital Rüdersdorf in Berlin. Each lasted one to two hours. Moderators (EK, JS) documented additional field notes, which were included in the analysis. Both sessions were digitally recorded, transcribed, and anonymized.

### Data analysis

Data analysis began concurrently with the interview phase. It was progressively extended to include all other data that was generated using the different methods of data collection. A coding framework was developed based on the collected material; key concepts and patterns were identified through grounded theory methodology [39]. The analysis was supported by MAXQDA software (VERBI GmbH, Berlin, Germany). The research team (EK, MS, JS) first conducted a thorough reading of each data set, wrote analytic memos, and engaged in a multi-step coding process. Coding followed three stages: (1) initial coding, (2) focused coding, and (3) theoretical coding [39]. Throughout the analysis, the research team met regularly to explore relationships between categories and to stimulate collective analytical thinking.

Following the completion of data analysis, the findings were presented in December 2023 to two representatives of the German national advocacy organization for carers of people with mental health conditions. The aim of this event was to contextualize the results from the perspective of carers and to generate input for the interpretation of findings in this publication, particularly with regard to implications for practice and health policy [38].

### Design

This article reports on the qualitative component of a larger project examining the effectiveness, costs, and implementation of intensive HT in Germany (“AKtiV study”) [25, 26]. Previous results from the overall study have been published elsewhere [27–33]. A sequential approach was used, with iterative switching between different methods of data collection and analysis [34]. First, the experiences of carers with HT were collected through qualitative interviews. In order to obtain more context-related data, the ethnographic method of participant diaries was used after initial evaluation [35, 36]. Subsequently, focus groups were conducted to address remaining knowledge gaps and to validate the findings [37]. Finally, a stakeholder consultation was conducted to plausibilize the findings, contextualize the results, and derive implications for practice and health policy [38]. The aim of the multi-method approach was to gain rich and ecologically valid insights. The data analysis was carried out according to the constructivist grounded theory paradigm as described by Kathy Charmaz [39]. The Ethics Committee of the Brandenburg Medical School (No. E-02-20200715) and the ethics committees of the participating study sites approved the study. The Consolidated Criteria for REporting Qualitative Research (COREQ) checklist was used to guide manuscript preparation [40] (see Supplementary File 1).

## Results

### Participants characteristics

Table 1 summarizes the sociodemographic characteristics of the study participants. Age was relatively evenly distributed across the categories from 25 to over 65 years. Gender was balanced between women and men. Most participants were highly educated, had completed vocational training, and were financially independent. The majority identified as white, of German background, and resided in metropolitan areas. More than half of the participants were partners or spouses of the service user (*n* = 15; 55.6%), and nearly three-quarters lived in the same household (*n* = 20; 74.1%), most commonly only with the service user (*n* = 12; 44.4%). Seven carers (25.9%) lived in separate households. Most carers reported daily contact with the service user (*n* = 23; 85.2%) and were present during treatment visits two to three times per week (*n* = 14; 51.9%). The vast majority had educated themselves about the service user’s mental health condition (*n* = 24; 88.9%). In contrast, only about one-third of carers had personal experience with psychiatric or psychotherapeutic treatment (*n* = 9; 33.3%), and just one carer participated in a self-help group for care (*n* = 1; 3.7%).

**Table 1:** Sociodemographic characteristics of the sample (*N* = 27)

Characteristics	Category	*n*	%
Age (years)	< 25	0	0
	25–35	6	22,2
	36–45	5	18,5
	46–55	4	14,8
	56–65	6	22,2
	> 65	6	22,2
Gender	male	13	48,1
	female	13	48,1
	diverse	1	3,7
Relationship to service user	spouse/partner	15	55.6
	parent	5	18.5
	other (i.e. child, sibling, state-employed caretaker)	7	25,9
Ethnicity	white	24	88,9
	person of color	1	3,7
	other	2	7,4
Immigration background^#^	yes	7	25,9
	no	20	74,1
Place of residence*	metropolitan	14	51,9
	urban	6	22,2
	rural	7	25,9
Highest certificate of education	university degree	15	55,6
	middle school diploma	9	33,3
	A-levels	3	11,1
Finished vocational training	yes	23	85,2
	no	4	14,8
Cost of living paid by	own salary	19	70,4
	pension	6	22,2
	partner, parent	2	7,4

^#^An immigration background is fulfilled when the subject, their parents or grandparents immigrated to Germany

*metropolitan: population > 1,000,000; urban: population 500,000–1,000,000; rural: population < 500,000

### Overview of qualitative findings

Three key concepts emerged from the analysis: ‘Contributions and Assumed Roles’, ‘Navigating Role Ambiguity and Expectations’, and ‘Support Needs and Challenges’. Table 2 presents the categories assigned to each key concept, which are described in detail in the sections below.

**Table 2:** Role of carers in psychiatric home treatment: key concepts and assigned categories

Key concepts	Categories
Contributions and assumed roles	Initiation of treatment Debriefing and emotional containment Providing information and translation Providing space for treatment Managing daily responsibilities
Navigating role ambiguity and expectations	Expected vs. actual involvement Role clarity and communication
Support needs and challenges	Acknowledgment and emotional support Reaching and exceeding limits

### Contributions and assumed roles

#### Initiation of treatment

The role of carers in HT was prominently reflected in the support they provided at the very outset of treatment. Because service users were at home, decisions about whether to pursue HT rather than hospital admission or another type of treatment often rested heavily on the carers’ influence. In some cases, carers had to make this decision independently during moments of an acute crisis. One mother, writing about her son who received HT, stated:

*He’s still very much attached to us and wants our feedback [mine and his father’s] about whether [HT] is the right thing. And we encouraged him that it’s worth trying it.* (mother; C01)

Caretakers often handled the logistics of admission and initiated contact with the clinic. Because HT is still less widely known among healthcare providers, some relatives reported encountering uncertainty or even a lack of awareness about HT within the medical system:

*I called a doctor I know - a friend of mine*,* our family doctor - and asked: What should we do now? And he then recommended the psychiatric outpatient clinic [a specialized service for people with severe mental illness in Germany]. I drove there directly with my daughter*,* just as she was. They then referred us to this HT and kindly registered us*,* and it started a few days later.* (father; C02)

#### Debriefing and emotional containment

Even during an ongoing HT episode, carers provided diverse forms of support, often directly related to the treatment process. One frequently described contribution was helping to absorb the considerable need for conversation that service users expressed after treatment appointments. Caregivers supported the processing, reflection, and emotional containment of topics that had been opened during therapeutic encounters. On the one hand, this allowed caregivers to emotionally synchronize more closely with the service user and to gain deeper insight into the treatment. On the other hand, the opportunity to provide support in this way was often described as personally rewarding:

*So it’s really nice to be there as a family member and to follow up and feel into it (How are you doing with this?) and also to give some encouragement and so on. It was also good for me and honestly not a burden*.

These conversations created space for service users to reflect on and talk about their HT experiences. In some cases, carers also had to absorb emotional crises that had been triggered by interactions with HT staff:

*Then she told me that the team member said things that weren’t true at all. She [the service user] had to correct him again and again. She was close to tears.*

#### Providing information and translation

Caregivers reported that they were able to provide crucial information during family or network meetings that contributed to the treatment process:

*As is usual with my daughter and me*,* we had a kind of joint conversation. We have a very good relationship. What she didn’t say*,* I knew. Then I said*,* ‘X*,* should I answer?’ And she said*,* ‘Oh yes*,* you go ahead.*

This may be especially important when cultural differences pose a challenge to communication. One service user had certain religious beliefs that stemmed from his upbringing in a different country, which could be misinterpreted as part of an ongoing psychosis. His friend and landlord was able to relate this to the psychologist:

*Due to the drug-induced psychosis*,* delusions then probably developed additionally*,* but these were very closely related to these religious ideas. And one must not consider these ideas to be a symptom of the psychosis or schizophrenia*,* so to speak… during HT*,* I also mentioned this to one of the psychologists*,* who was very grateful for the insight.*(C06).

By sharing their perspective during treatment conversations, carers contributed to a deeper clinical understanding of how the mental health crisis developed. This seemed helpful for HT staff and sometimes for the service user:

*Through me*,* the psychologist was able to take a different perspective and understand my sister’s [HT service user’s] problem in another way. (sister; C04)*

*I think it also helped my daughter move forward*,* as he seemed more open to considering my perspective during the therapy session. (father; C05)*

In one case, a relative served as a literal translator due to a language barrier between the HT team and the service user:

*In my case*,* my wife is Iranian*,* and I was always there as the translator at every home visit - otherwise it wouldn’t have worked. (male partner; C07)*

#### Providing space for treatment

Because caregivers and service users often shared the same household, participants frequently raised the question of where treatment should take place. In this context, carers actively contributed to creating a treatment environment by withdrawing during HT visits and ensuring a quiet atmosphere in the home while care was being provided. In some cases, carers even offered their own home as the location for HT, despite not living with the person in treatment, thereby enabling HT to take place in the first place:

*I live in X myself*,* and he’s way out in Y. Since the clinic was much closer to X*,* we arranged it so that he would basically live with me for a while and that the HT sessions would take place at my place. (mother; C07)*.

In rare cases, the provision of space also created strain for caregivers, particularly when rooms that were usually shared became unavailable due to treatment, or when space in general was limited:

*It was just sometimes a bit stressful because we live in a small apartment. And when my wife had her home visits*,* I wanted to give her some privacy*,* but sometimes I didn’t know where to go. So the living room was taken*,* and sometimes I just lay down in the bedroom and took a nap. (male partner; C08)*.

#### Managing daily responsibilities

The interviewed caregivers almost always described assuming everyday household tasks as part of their role, particularly during phases of acute illness and throughout the HT episode. One frequently mentioned responsibility was providing care for other family members in need of support, such as young children or elderly relatives. This form of care was implicitly or even explicitly expected by both the HT team and the service user. Several caregivers did not express concerns about taking on these additional responsibilities. However, they noted that the COVID-19 pandemic–and the associated shift to remote work–made this level of involvement possible in the first place. Without the ability to work from home, the increased demands of childcare might not have been manageable:

*I was waking up*,* taking the baby with me*,* and letting X have the treatment. It was fine because I was on parental leave for a couple of months*,* so my daily routine didn’t really change. If I had been working*,* it might have been a problem. (male partner*,* C09)*.

Some carers expressed frustration with these expectations, noting that the treatment period left them completely occupied:

*It was exactly during the time when the team was there that I couldn’t do anything else–I also had to keep our daughter entertained. (male partner; C10)*.

In addition to care responsibilities, caregivers often took on practical household tasks such as cleaning or grocery shopping. Daily cleaning, in particular, was perceived by some as more burdensome than usual due to the regular presence of staff in the home:

*If someone is in your home every day*,* you’re constantly cleaning. […] Then you vacuum*,* then you mop; after all*,* you want everyone to feel comfortable. And of course that was exhausting. (male partner; C10)*.

### Navigating role ambiguity and expectations

#### Expected vs. actual involvement

Caregivers discussed their involvement in HT in a variety of ways, with most reporting that they were included in some capacity. Nevertheless, many expressed a desire - often for different reasons - for more comprehensive engagement:

*A bit more intensive*,* a bit earlier*,* and again in the final phase would have been very important to us*,* but unfortunately that didn’t happen. (father; C05)*.

*I would have liked to be present more often*,* also in the therapy sessions. You learn from it yourself–how to do things*,* how to provide support. (male partner; C10)*.

While some participants justified their involvement based on a systemic understanding of illness, others explicitly declined participation due to challenging family relationships:

*Yes*,* it was important to me [to be involved] because the problems are systemic*,* as they say. So*,* my wife and I sometimes end up being the symptom bearers in our family system. (father; C08)*.

*No*,* we don’t really see ourselves as part of the therapy. I would have found that difficult*,* because the parent–daughter relationship isn’t exactly neutral*,* to put it very carefully. (mother; C28)*.

In some cases, service users themselves refused the involvement of their caretakers. One carer wished the tzeam had made greater efforts in such situations:

*We would have been included [by the HT team]*,* but the patient didn’t want it. For us*,* involving relatives is simply part of it–they could have found other ways to make it happen. (parents; C13)*.

#### Role clarity and communication

Regardless of the actual level of involvement, unclear communication from HT staff about the expected role of carers often led to confusion and uncertainty. Carers sometimes did not know how they should behave, whether they should take an active role, or when to step back:

*It’s very strange… because you’re not sure about your role. You don’t know: What role do I actually play in all of this? What is right*,* what is wrong*,* how should I approach things? (sister; C04)*.

*Yes*,* you were never quite sure: Should I get involved now or not? (father; C08)*.

Some linked this uncertainty to a lack of initiative from the team:

*I can imagine that many relatives*,* who don’t have much prior experience*,* have exactly this uncertainty and therefore don’t ask these questions. Maybe it should be up to the professionals to ask: ‘Is there something on your mind*,* something that’s making you uncomfortable?’ (male partner; C11)*.

Some caretakers also noted that certain expectations seemed to be in place without being explicitly stated:

*Interviewer: And what was expected of you–by the HT team*,* and by your sister? Carer: I definitely had to be reachable at all times. That was kind of an unspoken rule. (sister; C04)*.

Many expressed a desire for more direct communication and a structured approach to collaboration. Joint meetings involving the team, the service user, and caretakers at the start of HT were considered a way to clarify mutual expectations:

*I didn’t really feel involved. I was cautious–should I join in now*,* or are we doing things differently today? I missed having a plan or a direct invitation. That’s what I would have liked more. (male partner; C07)*.

*We had one network meeting*,* which I found very good*,* but maybe it would be better to have a joint session with relatives at the beginning to clarify: Are there expectations for the relatives at home? Or could they contribute more? (female partner; C11)*.

### Support needs and challenges

#### Acknowledgment and emotional support

Some carers expressed a need for greater support in their caregiving role. They wished that HT team staff would proactively reach out to them, offer emotional support, and provide space to address their own concerns. Several emphasized the importance of feeling acknowledged – both in their role within the social network of the service user and as individuals – which, in their view, did not always happen:

*I never really asked for it myself. […] But the opportunity also never arose on its own–like asking: ‘Would you like to talk about something?’ Or*,* ‘How is your workday*,* your private life*,* your household going? How does it work when certain areas fall away because a partner is less able to function or manage their own daily routine?’ (female partner; C03)*.

*They could have at least occasionally asked me how I was doing. It doesn’t have to be every day*,* but just being seen at all–as a relative–would have meant something. (female partner; C29)*.

When carers did feel that their support was acknowledged by HT team staff, they often described this recognition as encouraging and strengthening:

*It was simply about acknowledging what was happening at home*,* in the family–the burden–and saying: ‘Hats off to you for managing this.’ In that moment*,* I really appreciated hearing it from an outsider*,* from a neutral person. It gave me a bit of a boost*,* I must say. (female partner; C03)*.

Some caregivers said they would have felt better supported if they had received more information about available self-help resources:

*Something that was also on my mind […] was support services specifically for relatives. There are various self-help groups*,* forums*,* and so on. […] It would have been great to get that kind of information. (male partner; C07)*.

Others, however, did not want therapeutic support for themselves from the HT team, stating that they benefited most when the service user’s condition improved:

*I didn’t feel the need to have appointments with the HT team about my own concerns. I just wanted my husband to be helped. (female partner; C11)*.

#### Reaching and exceeding limits

Caregivers described situations in which they reached, or even exceeded, their limits during HT and no longer knew how to support the service user:

*For me*,* it was a very difficult situation because I wanted to be there for her*,* to be a strong support*,* but at the same time you also want to guide her in the right direction–in other words*,* not support her in the wrong way. But I didn’t know how. (sister; C04)*.

Others reached their limits due to the cumulative demands of everyday responsibilities combined with caring for the service user during treatment:

*I tried to do absolutely everything. By the end*,* I was truly exhausted. And yet*,* there was no other way. She couldn’t have stayed in the hospital either*,* because the language barrier would have left her much more isolated there. I tried to do the opposite–to maintain everyday life. But it was just too much. (male partner; C07)*.

Not all caregivers felt heard when they communicated these limits to the HT team. In some cases, the carer’s overload even led to the discontinuation of HT and the service user’s transfer to inpatient care:

*But no one really asked how I was doing with it. I had already said*,* two days before I took her to the clinic*,* that I couldn’t manage anymore. Then I was persuaded a bit to try again. And then I gave it one more day and realized–I just couldn’t do it. (mother; C07)*.

## Discussion

This study highlights the central yet multifaceted role of carers in acute psychiatric home treatment, illustrating both the breadth of their contributions and the complexity of the challenges they face. Carers were frequently integral to the initiation and maintenance of care, providing organizational support, emotional containment, and the practical conditions necessary for treatment delivery. However, these contributions were often accompanied by unclear role expectations, communication gaps, and varying degrees of recognition, which in some cases led to frustration, conflict, or overburdening. The findings suggest that while HT creates unique opportunities for sustained and meaningful involvement of carers, it also exposes them to increased demands if their role is insufficiently defined and supported.

### Relationship to previous research

The variety of functions assumed by carers in this study aligns closely with prior research on their roles in mental health care across settings [47]. These included initiating treatment in acute crises, providing organizational and emotional support, sustaining daily routines, and facilitating communication between service users and professionals. Some carers acted primarily as the patient’s personal coordinator, managing schedules and ensuring household stability. Others assumed the role of emotional anchors, providing continuous reassurance and containment between professional visits. Another group could be described as interpreters or advocates, translating the service user’s needs and perspectives to clinicians and, in some cases, negotiating cultural or linguistic differences. In contrast to inpatient contexts - where carers have often been described as occupying a peripheral position within a system dominated by institutional routines and professional control - our findings suggest that HT inherently places carers in a more central position [47]. Nevertheless, although they provided the framework for treatment, caregivers often felt insufficiently acknowledged and perceived themselves as “invisible,” much like in the inpatient setting [48]. This is partly due to the treatment being delivered within the home environment, where caregivers naturally hold logistical and emotional influence over the care process. Similar to the “gatekeeper” and “proxy” roles described in hospital-based psychiatric care [49], relatives in HT often acted as decision-makers during crises and as consistent providers of support over extended periods. This continuity, rarely possible in hospital settings, may strengthen the therapeutic alliance but also introduces unique demands on relatives. The bridging function carers perform - mediating between clinical teams and service users - has been recognised in other mental health settings [50, 51] and appears particularly relevant in HT, where effective collaboration hinges on the seamless integration of clinical and caregiver efforts.

A central finding of this study is the prevalence of unclear and inconsistently communicated role expectations for carers. Many participants reported uncertainty about their position in the treatment process, including when their active involvement was desired, what responsibilities they were expected to assume, and where the limits of their role lay [18]. This lack of clarity was compounded by the absence of formalised role discussions at treatment initiation, leaving caregivers to infer expectations from indirect cues or their own assumptions. Such ambiguity is not unique to HT; similar patterns have been described in inpatient and community mental health settings [22, 48], where role uncertainty can contribute to relational strain and reduce the effectiveness of collaboration. Without explicit negotiation of roles, carers may oscillate between overstepping and withdrawing, both of which can hinder therapeutic progress. The problem is further compounded when different stakeholders - service users, caregivers, and clinicians - hold divergent expectations. Structured, early-stage conversations to align these perspectives, coupled with ongoing communication throughout treatment, could help to establish a shared understanding and adapt roles as circumstances evolve.

The study also highlights the significant and often cumulative burden experienced by carers during HT. Balancing the emotional demands of supporting a person in an acute psychiatric crisis with the practical requirements of household management, employment, and other caregiving responsibilities placed many participants under sustained strain. These pressures were magnified in situations where professional support was perceived as insufficient or delayed, reinforcing the sense of being “left alone” with complex care tasks. There were no material or logistical support strategies within HT for carers to help manage daily responsibilities. This seems to be similar even in different sociopolitical contexts. A study analyzing mental health caregivers’ experiences across settings in Singapore reports that caregivers would greatly value employment-enabling and sustaining initiatives to help manage caregivers’ emotional and system challenges, as well as peer-support groups, life skills training, and public mental health awareness [52]. Broader literature has linked caregiver overload to increased risk of mental health problems, relationship difficulties, and reduced caregiving capacity [11, 12]. In the context of HT, the intensity and immediacy of home-based care can blur the boundaries between family life and clinical intervention, making it more difficult for caregivers to disengage and recover from the emotional demands of the role. Caregivers in our study often struggled to distinguish their own experiences from those of the service user, frequently framing their narratives around the latter’s well-being and difficulties. This tendency appears to reflect an implicit, and sometimes unconscious, expectation from both professionals and service users that caregivers should subordinate their own needs in order to provide support [53]. Importantly, the findings suggest that overburden is not solely a function of the quantity of tasks undertaken, but also of the perceived absence of recognition, guidance, and emotional support from the treatment team. Caregiver strain appears to be alleviated not merely by reducing the number of tasks, but by fostering meaningful and well-informed involvement - for instance, delegating practical or medical responsibilities that counteract feelings of helplessness [54]. Notably, very few caregivers in this study had experience with self-help groups: only one reported participation, while most were unaware such options even existed. Currently, support for caregivers across settings comprises a variety of psychoeducation interventions, self-help-groups, or mental support hotlines [55]. Many of these services are self-organized by caregiver associations [56]. Governmental financial support focuses mostly on the service users (e.g. sick pay, disability pension, etc.), although a nursing allowance that can be paid to informal caregivers is also provided. Proactive support strategies that acknowledge caregivers both in their role and as individuals - such as regular check-ins, the provision of psychoeducation, and information on peer or respite services - could mitigate these risks while sustaining the positive aspects of carer involvement.

### Strengths and limitations

To our knowledge, this is the first qualitative study to explore the perspectives of carers on their role in acute psychiatric home treatment in Germany. The inclusion of a relatively large and mostly diverse sample, drawn from multiple sites and using three different data collection formats, strengthens the robustness and transferability of the findings. There is a lack of diversity only in that the sample was overall well-resourced, financially independent and highly educated. This may influence the specific needs the participants expressed, though it also aligns with the findings of a systematic review on family perspectives within mental health interventions, which shows often more homogeneous samples (highly educated, middle income) since participants tend to self-select into the studies [23]. A study on mental health treatment in Bangladesh, on the other hand, focuses more on material challenges (cost, distance, etc.) as well as stigma and human rights abuses [57]. Although the qualitative design limits generalizability due to the lack of a sampling frame ensuring representativeness, data collection was continued until meaning (or theoretical) saturation was achieved, thereby strengthening the analytic robustness of the study [58]. The participatory elements of the study, involving both peers and representatives from the national family carers’ association, further enhanced its relevance and credibility. Nevertheless, potential selection bias cannot be excluded, given that participation required a certain level of openness, language proficiency, and interest in the topic. Moreover, the operational definition of a caregiver - requiring a “trusting relationship, close kinship, or personal closeness to the service user” - may have shaped participants’ perspectives and introduced additional bias. Interviews and analyses were conducted by the same researchers, which may have increased the risk of observer bias; however, iterative coding and reflexive group discussions during analysis were used to mitigate this risk. In addition, the study did not capture the perspectives of service users or HT staff, which represents an important limitation. However, this omission reflects the study’s explicit aim to investigate the carers’ perspective in depth.

### Implications for practice and research

The results point to an urgent need for systematic strategies to clarify and support the role of caregivers in HT. Establishing joint meetings early in treatment to discuss mutual expectations, desired levels of involvement, and potential boundaries could address much of the reported role ambiguity. Clinicians may benefit from training in carer engagement, particularly in navigating complex relationship dynamics and balancing the needs of service users with those of their caregivers [59]. Providing information on self-help resources, peer support, and other services tailored to relatives should be standard practice. From a research perspective, further studies should investigate the impact of different models of caregiver engagement in HT, ideally using participatory designs that capture the perspectives of all stakeholders, including service users, caregivers, and professionals. Developing and testing structured role negotiation tools could be a promising avenue to enhance clarity, reduce burden, and strengthen collaboration.

## Conclusions

Caregivers in acute psychiatric home treatment occupy a pivotal position that can enhance the reach, responsiveness, and contextual relevance of care. Their contributions - spanning crisis initiation, emotional containment, and the practical facilitation of treatment - are indispensable. Yet their potential is not fully realized when roles remain undefined, communication is inconsistent, or support mechanisms are lacking. The evidence from this study underscores the importance of integrating structured role clarification and tailored support into the design of HT services. Doing so could not only strengthen therapeutic collaboration but also protect the well-being of carers, thereby ensuring the sustainability of their involvement over time. Beyond the immediate context of HT, these findings may have implications for other forms of community-based psychiatric care, particularly those that rely heavily on caregiver participation. Future research should examine the effectiveness of formalised role negotiation tools and support interventions, ideally through participatory and longitudinal designs that capture changes in caregiver involvement over the course of treatment.

## Supplementary Information

Below is the link to the electronic supplementary material.

Supplementary Material 1

Supplementary Material 2

## Data Availability

The datasets generated and/or analyzed during the current study are not publicly available due to the sensitive and confidential nature of the data, which involve identifiable information from family members of patients. Data are available from the corresponding author on reasonable request and subject to appropriate ethical approvals.
