# Supplementary material for: Exploring the role of carers in psychiatric home treatment: qualitative multi-method study
Source: BMC Psychiatry. 2026 Apr 1;26:294. doi: 10.1186/s12888-026-08018-9 (PMC13063616; doi:10.1186/s12888-026-08018-9)
Supplement: Supplementary file 1 — Supplementary Material 1 [file 12888_2026_8018_MOESM1_ESM.docx]

**Supplemental file 1**

**Consolidated Criteria for Reporting Qualitative Research (COREQ) Checklist**

| **Item No** | **Guide Questions/Description** | **Reported on Page #** |
| --- | --- | --- |
| **Domain 1: Research team and reflexivity** | |  |
| **Personal Characteristics** | |  |
| 1 | Interviewer/facilitator: Which author/s conducted the interview or focus group? | Pg 2 |
| 2 | Credentials: What were the researcher’s credentials? E.g., PhD, MD | Title page |
| 3 | Occupation: What was their occupation at the time of the study? | Pg 3 |
| 4 | Gender: Was the researcher male or female? | Pg 3 |
| 5 | Experience and training: What experience or training did the researcher have? | N/A |
| **Relationship with participants** | |  |
| 6 | Relationship established: Was a relationship established prior to study commencement? | Pg 2 |
| 7 | Participant knowledge of the interviewer: What did the participants know about the researcher? | Pg 2 |
| 8 | Interviewer characteristics: e.g., bias, assumptions, reasons and interests in the research topic | N/A |
| **Domain 2: Study design** | |  |
| 9 | Methodological orientation and theory: e.g., grounded theory, ethnography, phenomenology, etc. | Pg 3 |
| **Participant Selection** | |  |
| 10 | Sampling: How were participants selected? | Pg 2 |
| 11 | Method of approach: How were participants approached? | Pg 2 |
| 12 | Sample size: How many participants were in the study? | Pg 2-3 |
| 13 | Non-participation: How many refused or dropped out? Reasons? | N/A |
| **Setting** |  |  |
| 14 | Setting of data collection: Where was data collected? e.g., home, clinic | Pg 3 |
| 15 | Presence of nonparticipants: Was anyone else present? | Pg 2 |
| 16 | Description of sample: Demographics, etc. | Pg 3-5 |
| **Data Collection** | |  |
| 17 | Interview guide: Were guides provided or pilot tested? | Pg 2-3 |
| 18 | Repeat interviews: Were repeat interviews carried out? | Pg 2 |
| 19 | Audio/visual recording: Were recordings used? | Pg 3 |
| 20 | Field notes: Were notes taken during or after interviews? | Pg 2-3 |
| 21 | Duration: What was the duration of interviews or focus groups? | Pg 2-3 |
| 22 | Data saturation: Was saturation discussed? | Pg 2-3 |
| 23 | Transcripts returned: Were transcripts returned to participants? | N/A |
| **Domain 3: Analysis and findings** | |  |
| 24 | Number of data coders: How many coders worked on the data? | Pg 3 |
| 25 | Description of coding tree | Pg 5 |
| 26 | Derivation of themes: Were themes pre-set or derived? | Pg 3 |
| 27 | Software: What software was used? | Pg 3 |
| 28 | Participant checking: Did participants check or comment on the findings? | Pg 3 |
| **Reporting** | |  |
| 29 | Quotations presented: Were quotes used and identified? | Pg 6–10 |
| 30 | Data and findings consistent: Was the interpretation consistent with the data? | Pg 5–10 |
| 31 | Clarity of major themes | Pg 5–10 |
| 32 | Clarity of minor themes | Pg 5–10 |
