# Supplementary material for: Exploring the role of carers in psychiatric home treatment: qualitative multi-method study
Source: BMC Psychiatry. 2026 Apr 1;26:294. doi: 10.1186/s12888-026-08018-9 (PMC13063616; doi:10.1186/s12888-026-08018-9)
Supplement: Supplementary file 2 — Supplementary Material 2 [file 12888_2026_8018_MOESM2_ESM.docx]

**Supplemental file 2**

**Interview guide**

Overarching research questions:

1. How do caregivers experience outreach acute psychiatric treatment (HT)?

2. What role do caregivers play in the home treatment setting?

| **Introduction** | |
| --- | --- |
| **Personal introduction** | - *Thank you for taking the time to talk to us.* - *Introduction of the interviewer* - *Explanation of the study Aktiv: we are a collaborative research project in which people with their own experience of psychiatric crises, ethnologists, doctors, and doctoral students work together* |
| **Background, aims, structure of the interview** | *In the Aktiv study, we are trying to find out more about how service users and caregivers experience acute outreach psychiatric treatment.*  *The interview should last between 60 and 90 minutes, depending on what is needed.*  *We can take a break at any time. Let us know if it gets tiring.* |
| **Clarifications in advance** | - *Emphasize that we are interested in positive and negative feedback. Everything will be anonymized & what you tell us will not be shared (especially not with staff).* |

| 1. **INTRODUCTORY QUESTIONS** | | |
| --- | --- | --- |
| **Access, information & expectation** | 1. **Looking back, how was HT for you as a caregiver?**    1. *What did you expect from HT?*    2. *To what extent were you involved in the decision for HT as a caretaker?*    3. *What hopes did you have? What fears did you have?*    4. *What was helpful about HT for you or other people around you?*    5. *To what extent did you or other people feel relieved or unburdened by HT?*    6. *How satisfied or dissatisfied are you with the course of treatment and its outcome?*   *-------------------------------------------------------------------------------*   - *How was that for you?* - *How did you feel about it?* - *How did it affect you?* | |

| 1. **MAIN QUESTIONS** | |
| --- | --- |
| **Involvement, treatment**  **processes and continuity** | 1. ***How were you involved in HT (or not)?***    1. ***To what extent were you involved in HT?*** *Were you involved in any conversations? How were these structured? Were there individual conversations with you and if so, how did they go? What topics were discussed?*   *To what extent did the HT team influence the relationship with the service user?*   - 1. ***To what extent did you want to be involved in HT?*** *How important was it to you to be involved? How did you feel about being involved or participating in the treatment, or not being involved or participating in the treatment?*   2. ***Did network meetings take place?*** *Who was involved in the network meetings? How often did they take place? Which other people from the user's environment were involved in HT?* - *How was that for you?* - *How did you feel about it?* - *How did it affect you?* |

| **Importance of setting and space** | 1. ***What was it like for you to have***   ***employees come to your home?***   - 1. ***How did the employees behave in your home?*** *How did employees deal with your privacy? What rules should employees observe?*   2. ***To what extent has the presence of staff influenced/disrupted your everyday life and that of the user?*** *Did you do things differently because staff came to your home? To what extent were the times at which the team came to your home suitable? Were they coordinated with you? If NO: what would have been more suitable and why?*   3. *Do you think that the employees were recognizable to outsiders (e.g. your neighbors) as clinic employees and how did you feel about this?*   4. *Were there moments when contact with employees was too close or too distant for you? (spatially but also emotionally)*   5. *To what extent was the duration of the treatment appropriate?* |
| --- | --- |

| **Assumed and assigned roles** | 1. ***What was expected of you as a caretaker?***     1. *What did the service user expect from you during the treatment period?*    2. *To what extent did the service user expect you to get involved?*    3. *What did the HT team expect of you as a caretaker?*    4. *To what extent did these expectations match what you were able to do?* |
| --- | --- |

| **Comparison, advantages and disadvantages** | 1. ***How did you experience HT compared to (partial) inpatient treatment?***    1. *What was different/better/worse when the service user was treated as an inpatient?*    2. *What are the advantages and disadvantages of HT from your personal point of view?*    3. *To what extent would you have found it safer/less safe if the service user had been temporarily hospitalized? (e.g. in threatening situations)* |
| --- | --- |

| **Possibilities for improvement** | - *What did you find helpful (or annoying, stressful, etc.) in relation to HT?* - *What was too much for you? What was missing?* - *If you needed help in a crisis yourself, would you make use of HT? Why yes/no?* - *If a relative or person you care for needed help again, would you participate in the treatment process as a caretaker during HT?* - *If you could change HT, what would you change?* |
| --- | --- |

| **Conclusion, Sociodemographics and Farewell** | |
| --- | --- |
| **Additional topics** | *(1)  Are there any other topics you would like to address?*  *(2)   Or questions?* |
| **Sociodemographics** |  |
| **Conclusion and farewell** | *Thank you very much for your time.* |

**Participant diary writing prompt**

You are a relative, partner, friend, roommate or have a similarly close relationship with a person who is receiving daily treatment at home due to an acute mental health crisis. Such a crisis can change the everyday lives of everyone involved. You have the unique role of probably not being personally afflicted, but by your proximity to the person being treated, seeing a lot of the treatment, sharing in the experience and probably taking on some responsibility. Please answer the following questions in your diary:

*How is the service user’s treatment for you? What is it like for you during the visits and in between?*

*Has your everyday life changed as a result of HT compared to the time before treatment?*

*(How) are you involved during treatment? How does that feel?*

*And - if everything were possible - how could HT be improved in regards to your situation?*

Pick up your diary whenever you think of something about the service user’s treatment or when you have a quiet moment. Write down everything you observe, your thoughts and feelings about it, your comments, ideas and questions. It can also be short entries, the important thing is to write every day if possible. You can reflect on how you feel about HT, the HT team and the service user, and perhaps the diary can become a kind of personal companion during the treatment. We hope you enjoy writing!
